# *MicroRNA-183* suppresses cancer stem-like cell properties in EBV-associated nasopharyngeal carcinoma

**DOI:** 10.1186/s12885-016-2525-5

**Published:** 2016-07-19

**Authors:** Chartia Ching-Mei Cheung, Samantha Wei-Man Lun, Grace Tin-Yun Chung, Chit Chow, Carman Lo, Kwong-Wai Choy, Kwok-Wai Lo

**Affiliations:** Department of Anatomical and Cellular Pathology, State Key Laboratory in Oncology in South China, Prince of Wales Hospital, The Chinese University of Hong Kong, Shatin, N.T. Hong Kong; Li Ka Shing Institute of Health Science, The Chinese University of Hong Kong, Shatin, N.T. Hong Kong; Department of Obstetrics and Gynecology, Prince of Wales Hospital, The Chinese University of Hong Kong, Shatin, N.T. Hong Kong

**Keywords:** Nasopharyngeal carcinoma, Epstein-Barr virus, microRNA, Cancer stem-like cells, NOTCH

## Abstract

**Background:**

Nasopharyngeal carcinoma (NPC) is an Epstein-Barr virus (EBV)-associated epithelial malignancy that exhibits distinct geographical and ethnic prevalence. Although the contemporary therapeutic approach of radio-/chemotherapy provides excellent results for patients with early-stage disease, it is far from satisfactory for those with disease remission and distant metastasis. Promising therapeutic strategies for advanced and relapsed NPC are still lacking. We recently identified and characterized a cancer stem-like cell (CSC) subpopulation in NPC that appeared to play an important role in tumor progression. Microarray analysis revealed downregulation of several stemness-inhibiting miRNAs in these CSC cells. Among these miRNAs, *miR-96* and *miR-183* showed the highest fold change and were selected to elucidate their role in repressing NPC CSC properties.

**Methods:**

*MiR-96* and *miR-183* expression in NPC CSCs was detected by qRT-PCR. Transient and stable transfection was performed in EBV-positive NPC C666-1 cells to examine the effects of ectopic expression of *miR-96* and *miR-183* on repressing cell growth and CSC properties. Anchorage-dependent (colony formation) and anchorage-independent (tumor sphere formation) growths of these *miR-96* and *miR-183* expressing cells were determined. Expression of multiple CSC markers and related molecules were accessed by flow cytometry and Western blotting. The tumorigenicity of the stable *miR-96-* and *miR-183*-transfected NPC cells was examined in an in vivo nude mice model.

**Results:**

Downregulation of *miR-96* and *miR-183* was confirmed in NPC spheroids. Using transient or stable transfection, we showed that ectopic expression of *miR-96* and *miR-183* suppressed cell growth and tumor sphere formation in NPC. Reduced NICD3 and NICD4 in *miR-96*- and *miR-183*-expressing NPC cells suggests the involvement of the NOTCH signaling pathway in their tumor suppressive function. Finally, we showed that the tumorigenicity of cells stably expressing *miR-183* was significantly inhibited in the in vivo nude mice model.

**Conclusions:**

*miR-183* is a tumor-suppressive miRNA in EBV-associated NPC. Its abilities to suppress CSC properties in vitro and effectively reduce tumor growth in vivo shed light on its role as a potential therapeutic target.

**Electronic supplementary material:**

The online version of this article (doi:10.1186/s12885-016-2525-5) contains supplementary material, which is available to authorized users.

## Background

Nasopharyngeal carcinoma (NPC) is an Epstein-Barr virus (EBV)-associated epithelial malignancy that exhibits distinct geographical and ethnic prevalence [1, 2]. Although the contemporary therapeutic approach of radio-/chemotherapy provides excellent results for patients with early-stage disease, it is far from satisfactory for those with disease remission and distant metastasis, which are highly fatal. [3]. Identification of promising therapeutic targets for patients with advanced disease is urgently needed. Despite continuous efforts in NPC research, our understanding of the mechanisms that regulate tumor progression is limited. In the past decade, the cancer stem-like cell (CSC) subpopulation was proposed to play a critical role in local relapse and metastasis in human cancers [4, 5]. These CSCs have the ability to self-renew, differentiate, and sustain propagation and are commonly unresponsive to conventional treatments [6]. Targeting of the CSC subpopulation and suppression of the properties of CSCs are believed to enhance the efficacy of radiotherapy and chemoradiotherapy [6]. In our earlier study, we identified and characterized an NPC CSC subpopulation that is suggested to be a potential culprit for the metastasis of this EBV-associated malignancy [7, 8]. Using microarray analysis, we revealed a number of differentially expressed genes in transcription regulation (e.g., *FOXN4* and *GLI1*), immune response (*CCR7*), and transmembrane transport (e.g., *ABCC3* and *ABCC11*) in the spheroids [7]. Aside from these cellular genes, microRNA (miRNA) microarray analysis also identified a number of differentially expressed miRNAs in the NPC CSCs. MiRNAs are small (approximately 22 nucleotides) non-coding RNAs that regulate gene expression [9]. Since the elucidation of the roles of miRNAs in developmental processes, studies have focused on their involvement in cancer [9, 10]. Interestingly, certain miRNA clusters, such as the *miR-183-96-182* cluster, play crucial roles in regulating stemness properties and drug resistance in cancer cells [11]. Wellner et al. showed that overexpression of *miR-183, miR-203*, and *miR-200c* decreases the sphere-forming capacity of pancreatic cancer cells [12]. It has been suggested that repression of these stemness-inhibiting miRNAs maintains the stem cell phenotype and is implicated in cancer progression [13–16]. Among the differentially expressed miRNAs identified in NPC spheroids, several stemness-inhibiting miRNAs including *miR-96* and *miR-183* were downregulated in the NPC CSCs. In the present study, we confirmed that *miR-96* and *miR-183* have the highest fold changes. We then performed a functional study to elucidate whether *miR-96* and *miR-183* are NPC tumor suppressors that repress CSC properties. Our findings demonstrated that the ectopic expression of *miR-96* and *miR-183* suppressed the colony- and sphere-forming ability of NPC cells in vitro. However, only NPC cells stably expressing *miR-183* could inhibit tumor formation in vivo in a nude mice model. *MiR-183* has a potent effect on the suppression of CSC properties in vitro and in vivo and may play a contributory role in NPC tumorigenesis.

## Methods

### Cell culture and transfections

An EBV-positive NPC cell line C666-1 was used in this study [17]. It was cultured in RPMI-1640 (Sigma-Aldrich, St. Louis, MO, USA) supplemented with 10 % fetal bovine serum (Invitrogen, Carlsbad, CA, USA). Tumor spheres (anchorage-independent growth) were cultured as previously described [7]. C666-1 cells were transiently transfected with *miR-96, miR-183,* Ambion® anti-miR™ miRNA inhibitors, or negative controls (Ambion, Austin, TX, USA) by Lipofectamine™ 2000 (Invitrogen) according to the manufacturer’s instructions. C666-1 cells stably overexpressing miRNA were generated by lentiviral transfection with a vector expressing *miR-96* or *miR-18* and a miR-negative control vector according to the manufacturer’s protocol (Lenti-miR™ microRNA precursor clones, SBI System Biosciences, Palo Alto, CA, USA). Successfully transfected cells were identified and confirmed by the expression of green fluorescence protein.

### Microarray analysis

Total RNA was extracted from sphere-forming and parental C666-1 cells and subjected to microarray analysis (Agilent Technologies Inc., Santa Clara, CA, USA) as described previously [7]. Aberrantly expressed miRNAs detected in the array were then subjected to quantitative reverse transcription and polymerase chain reaction (qRT-PCR) for subsequent validation.

### qRT-PCR analysis

Total RNA from each treatment group was extracted using TRIZOL® reagent (Invitrogen). qRT-PCR using SuperScript™ III Reverse Transcriptase (Invitrogen) was performed for the detection of 5 s for data normalization. For detection of miRNAs, reactions were carried out with TaqMan MicroRNA Assays (Life Technologies / Thermo Fisher Scientific, MA, USA) according to the manufacturer’s instructions. The assays employed pre-designed, target-specific stem-loop reverse transcription miRNA primers (Thermo Fisher Scientific) for the mature miRNAs. All qRT-PCRs were performed in triplicates on an ABI 7500 real-time PCR system (Applied Biosystems, Foster City, CA, USA) as instructed by the manufacturer.

### Western blotting

The expression of various proteins in the miRNA-expressing and control NPC C666-1 cells was detected by Western blotting. The antibodies against SOX2 (Abcam, Cambridge, MA, USA), OCT4 (Santa Cruz Biotechnology, Inc., Santa Cruz, CA, USA), BMI1 (Millipore, Billerica, MA, USA), NOTCH3/NICD3 (Orbigene, San Diego, CA, USA), NOTCH4/NICD4 (Orbigene), CYCLIND1 (Labvision/Invitrogen), and ACTIN (Santa Cruz) were used. In brief, C666-1 cells transfected with miRNAs or with vector control were harvested and lysed with cold radioimmunoprecipitation assay buffer (150 mM NaCl, 5 mM EDTA, 50 mM Tris, 1 % NP-40, 0.5 % sodium deoxycholate, 0.1 % sodium dodecyl sulphate, and protease phosphatase inhibitor cocktail). An equal amount of total protein from each sample was resolved on SDS-polyacrylamide gel and transferred onto nitrocellulose membrane (GE Healthcare UK Ltd., Little Chalfont, UK). The membrane was blocked with 5 % non-fat milk and was incubated with primary antibodies followed by the corresponding secondary antibodies. Protein expression was visualized using chemiluminescence exposed on X-ray films (GE Healthcare). β-actin was used as an internal loading control in the analysis.

### Fluorescence-activated cell sorting (FACS) analysis

Single-cell suspensions were rinsed twice and resuspended in phosphate-buffered saline (PBS) (10^5^ cells/100 μl) and subjected to FACS analysis as previously described [7]. In brief, for intracellular staining, cells were fixed in 70 % ethanol and subjected to 2 % human serum blocking of non-specific epitopes. Fluorescence-conjugated anti-CD44 (BD Biosciences, San Jose, CA, USA), anti-SOX2 and anti-BMI1 (R&D Systems, Minneapolis, MN, USA), and anti-OCT4 and anti-NANOG (ebiosciences, San Diego, CA, USA) antibodies were used in this study. Respective IgG isotypic controls were included in the experiment. At least 10,000 cells were acquired for each test sample and analyzed with a BD FACSCalibur flow cytometer (Becton Dickinson, Franklin Lakes, NJ, USA) and Flowjo software (Treestar/ Flowjo, LLC, Ashland, OR, USA).

### Colony formation assay

Cells (1 × 10^3^) with or without miRNA overexpression were seeded onto 100-mm^2^ plates and cultured for 7–10 days. They were then washed with PBS, fixed in methanol for 10 min, and stained with Giemsa stain. Experiments were performed in triplicate, and colonies with at least 50 cells (C666-1 cells of ≥ 1 mm in diameter) were manually counted under a stereomicroscope [18] and compared between the different groups.

### *In vivo* tumorigenicity assay in nude mice

To evaluate the tumorigenic potential, 2x10^6^ C666-1 cells with or without miRNA overexpression were subcutaneously inoculated into the flank of female BALB/c nude mice (nu/nu) (3 mice/group). The mice were inspected daily for tumor formation. After 4 to 12 weeks, the mice were killed by cervical dislocation and the tumors retrieved. All experimental procedures were approved by the Animal Ethics Committee of the Chinese University of Hong Kong.

### Statistical analysis

Tests were repeated at least three times independently for statistical calculations. Unless otherwise stated, an unpaired *t*-test was used for statistical analysis of the data. Statistical significance (*P* < 0.05) was determined by PRISM5 (GraphPad Software, Inc., La Jolla, CA, USA) and presented graphically as mean ± standard error (SE).

## Results

### Aberrantly downregulated expression of *miR-96* and *miR-183* in NPC CSCs

**Fig. 1:**
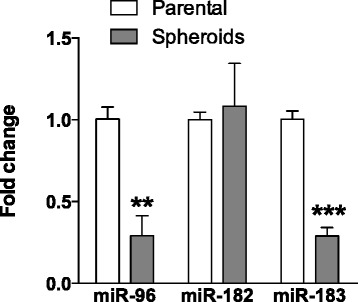
Down-regulation of *miR-96* and *miR-183* in NPC sphere-forming cells. The expression of *miR-96*, *miR-182*, and *miR-183* in sphere-forming and parental C666-1 cells was determined by qRT-PCR. The histogram shows the fold changes of miRNA expressions between sphere-forming and parental C666-1 cells. Expression of *miR-96* and *miR-183* was significantly decreased in sphere-forming C666-1 cells. No significant difference in *miR-182* expression was found. Student’s *t*-test was used to determine statistical significance between the two groups (*n* = 3, **P < 0.01, ***P < 0.001)

The miRNA expression profiles of NPC CSCs and parental C666-1 cells were determined by microarray analysis following our previous study [7]. The aberrantly downregulated miRNAs identified in NPC CSCs are summarized in Additional file 1: Figure S1A. Several downregulated miRNAs including *miR-200a* and *miR-203* were previously reported to modulate CSC properties in NPC cells [19, 20]. Among the downregulated miRNAs in the microarray analysis, we found that *miR-96* and *miR-183* showed the highest fold changes in NPC CSCs. Furthermore, the function of these miRNAs in NPC has not been explored. Thus, this study focused on elucidating the role of this cluster of miRNAs in NPC. As shown in Fig. 1, we confirmed that only the expression of *miR-96* and *miR-183*, but not *miR-182*, was significantly downregulated in NPC CSCs when compared to parental C666-1 cells. Significant downregulation of *miR-200a* and *miR-203* expression was also detected in NPC CSCs (Additional file 1: Figure S1B).

### Transient *miR-96* and *miR-183* expression inhibits colony formation and anchorage-independent growth in vitro

**Fig. 2:**
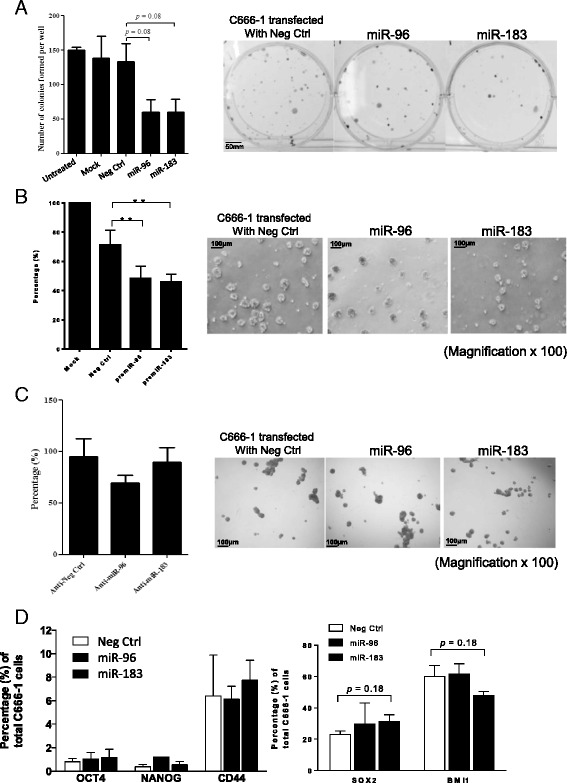
Transient expression of *miR-96* and *miR-183* inhibits colony and tumor sphere formation in NPC cells. The effects of transient overexpression of *miR-96* and *mi-183* on NPC cell growth were examined. **a** Colony formation assay of C666-1 cells with *miR-96* and *miR-183* overexpression. The numbers of colonies formed by C666-1 cells transfected with *miR-96* and *miR-183* were obviously lower than those of the negative control (*n* = 3, *P* = 0.08). **b** Tumor sphere formation in C666-1 cells expressing *miR-96* and *miR-183* was significantly inhibited when compared to that of the negative control (*n* = 3, **P < 0.01). Representative photos of tumor spheres are shown in the *right panel* (magnification × 100). **c** No change in tumor sphere formation in the C666-1 cells co-transfected with *miR-96* or *miR-183* and the corresponding anti-miR inhibitors was observed when compared to negative controls (*n* = 3, all *P* > 0.05). Representative photos of tumor spheres are shown in the *right panel* (magnification × 100). **d** Tumor cells expressing OCT4, NANOG, CD44, SOX2, and BMI1 were quantified by flow cytometry analysis. The histogram shows the percentage of cells expressing these proteins in C666-1 cells transfected with *miR-96* and *miR-183* and that of the negative control. No significant changes in OCT4, NANOG, CD44, SOX2, and BMI1 expression were observed in the cells transfected with *miR-96* and *miR-183* (*n* = 3, all *P* > 0.05). Student’s *t*-test was used to determine statistical significance between the two groups

To evaluate the function of *miR-96* and *miR-183* in NPC, the effects of the ectopic expression of these miRNAs on cancer stem-like properties were studied in transiently transfected C666-1 cells. Although the overexpression of *miR-96* and *miR-183* showed an obviously suppressive effect on anchorage-dependent C666-1 growth (Fig. 2a, *P* = 0.08), a significant inhibition in anchorage-independent growth was observed (Fig. 2b, *P* < 0.01). As shown in Fig. 2c, the inhibitory effect of *miR-96* and *miR-183* overexpression on anchorage-independent growth was negated by anti-*miR-96* and anti-*miR-183* expression, respectively.

Despite the growth inhibitory effects observed above, no aberrant expression of pluripotency-related stem cell transcription factors OCT4, SOX2, and PcG protein BMI1 and cell surface marker CD44 were detected in the NPC cells expressing *miR-96* or *miR-183* by flow cytometry when compared with that of the negative control (Fig. 2d, all *P* > 0.05).

### NPC cells stably expressing *miR-96* and *miR-183* show reduced tumor sphere-forming capacities

**Fig. 3:**
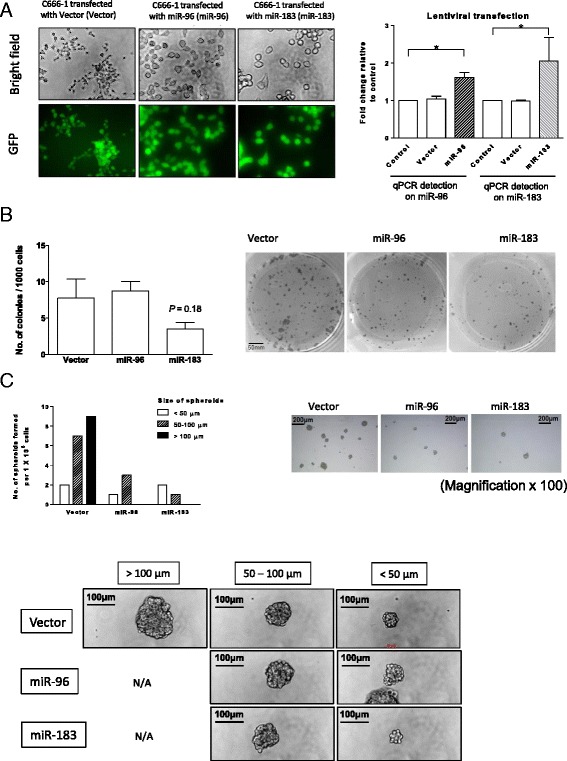
Effects of stable overexpression of *miR-96* and *miR-183* in C666-1. **a** C666-1 cells stably expressing *miR-96* and *miR-183* were established by lentiviral transfection. *Left panel*: Transfection efficiency of the transfected cells expressing green fluorescence protein (GFP) was determined under microscopy. Representative photos show that over 90 % of the cells are visualized in green. *Right panel*: The expression of *miR-96* or *miR-186* in the stably transfected cells was confirmed by qRT-PCR. Histograms confirmed elevated expressions of *miR-96* and *miR-183* in the respective cells stably transfected with *miR-96* and *miR-183* (*n* = 3, all *P < 0.05). The effects of *miR-96* and *miR-183* overexpression in NPC cells were assessed by their (**b**) colony-forming and (**c**) sphere-forming capacities. **b** The histogram shows the number of colonies formed in the C666-1 cells expressing *miR-96* or *miR-183* compared to that of the vector control. The colony number of C666-1 cells expressing *miR-183* is obviously lower than that of the control (*n* = 3, *P* = 0.18). However, no aberration was observed in colony formation between cells stably transfected with *miR-96* and those with the vector. Representative photos of the colonies are shown in the right panel. **c** Both the number and size of the tumor spheres were reduced in the C666-1 cells expressing *miR-96* and *miR-183*. The number of tumor spheres in the C666-1 cells stably expressing *miR-96* and *miR-183* and the vector control is illustrated in the histogram according to the sizes of the tumor spheres formed (<50 μm, 50–100 μm, > 100 μm) (mean data from 6 wells of a 6-well plate per group). C666-1 cells stably expressing *miR-96* and *miR-183* failed to form tumor spheres of diameter larger than 100 μm. Representative photos of the tumor spheres are shown in the *right panel* (magnification × 100). *Lower panel*: Representative photomicrographs reveal the difference in size of the spheres between each treatment group

To further examine the effect of overexpression of *miR-96* and *miR-183* on NPC CSCs, lentiviral-based vectors expressing *miR-96* and *miR-183* were used to establish stably transfected C666-1 cells. As shown in Fig. 3a, lentiviral transfection of the miRNAs produced over 90 % efficiency in C666-1 cells. The NPC cells stably expressing *miR-96* and *miR-183* were indicated by the expression of green fluorescent protein. Significantly increased *miR-96* and *miR-183* expressions were detected in the stably lentiviral-transfected C666-1 lines by qRT-PCR when compared to that of non-transfected cells (Fig. 3a, *P* < 0.05).

C666-1 cells with stable *miR-183* overexpression showed an observable decrease in the number of colonies formed when compared to those transfected with vectors (Fig. 2b, *P* = 0.18). However, no aberration was observed in colony formation between cells stably transfected with *miR-96* and those with the vector only. Interestingly, a decrease in the number of spheres formed was observed in cells stably expressing *miR-96* or *miR-183* when compared to those with the vector (Fig. 3c). Stable overexpression of these two miRNAs completely abolished the formation of tumor spheres larger than 100 μm in diameter (Fig. 3c).

### *miR-96* and *miR-183* repress stemness transcription factors and NOTCH signals

**Fig. 4:**
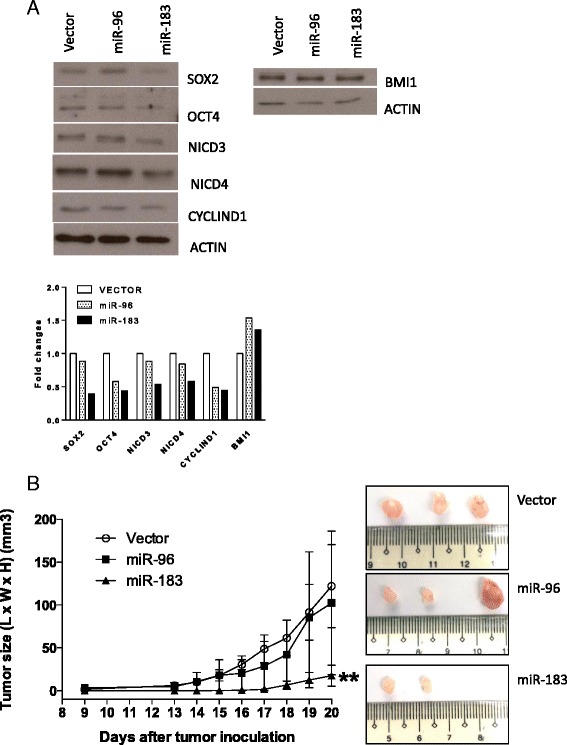
Overexpression of *miR-183* inhibits stemness and tumorigenic potential in C666-1 cells. **a** The expression of stem cell transcription factors NICD3, NICD4, and CYCLIND D1 in the C666-1 cells stably expressing *miR-96* and *miR-183* was detected by Western blot. Reduced expression of SOX2, OCT4, NICD3, and NICD4 was found in the C666-1 cells with overexpression of *miR-96* or *miR-183*. Similar BMI and CYCLIN D1 expression was observed in the C666-1 cells stably expressing *miR-96* and *miR-18* and the vector control. Beta-actin was used as the internal loading control. The results were quantified and the histogram shows the fold changes between stable transfected cells and the vector control. **b** The tumorigenic potential of the C666-1 cells stably expressing *miR-96* and *miR-183* was assessed by tumor growth in an in vivo nude mice model. Significant inhibition of tumor formation was observed in the C666-1 cells stably expressing *miR-183* (**P < 0.01) but not in the cells stably expressing *miR-96*. Photos reveal the appearances and sizes of the tumors in each treatment group. Student’s *t*-test was used to determine statistical significance between the two groups (*n* = 3, **P < 0.01)

Western blotting showed reduced expression of stem cell transcription factors SOX2 and OCT4 in C666-1 cells stably expressing *miR-96* or *miR-183* (Fig. 4a). Similar BMI1 expression was noted in the stably transfected cells and vector controls (Fig. 4a). In our previous study, overexpression of CYCLIN D1 and activation of NOTCH signaling were shown to play a critical role in the cell growth and survival of NPC [21]. In the present study, we found that the expression of NICD3 and NICD4, two surrogate markers for activated NOTCH signaling [22], were downregulated in the NPC cells overexpressing *miR-96* or *miR-183* (Fig. 4a). However, no reduced expression of CYCLIN D1 was observed in the stably miRNA-transfected cells (Fig. 4a).

### miR-183 inhibits in vivo NPC tumor formation

In Fig. 4b, the C666-1 cells stably expressing *miR-183* showed a significant reduction in in vivo tumor formation when compared to those transfected with the vector in the nude mice model (*P* < 0.01). However, no significant changes were observed in the NPC cells stably expressing *miR-96* (Fig. 4b).

## Discussion

In our earlier study, we identified and characterized a CSC subpopulation in EBV-positive cells [7, 8]. In the present study, using microarray assay and qRT-PCR analysis, we revealed that *miR-96* and *miR-183* are highly repressed in NPC CSCs (Fig. 1 and Additional file 1: Figure S1A). Although *miR-96* and *miR-183* have been suggested to be oncogenic, promoting tumor cell migration and invasion in various cancers [23–26], studies have also shown that they play a critical role in epithelial-mesenchymal transition, via inhibition of cell migration and invasion, downstream of the p53-p21 pathway [27]. Inhibition of tumor cell migration and invasion by these miRNAs was also reported in osteosarcoma and pancreatic and gastric cancers [15, 16, 28, 29]. With this study, we provide the first evidence that *miR-183* exerts tumor-suppressive effects on NPC by repressing CSC properties. Overexpression of *miR-183* in NPC C666-1 cells significantly inhibited cell growth and tumor formation in vivo (Figs. 2, 3 and 4). The downregulation of stemness markers and NICDs by *miR-183* also supports its function in the inhibition of NPC CSC properties (Fig. 4a). Recently, Tang et al. showed that *miR-183* expression negatively correlates with lymph node status in primary NPC [30]. The correlation is likely to be due to the suppressive role of *miR-183* in NPC CSCs. Emerging evidence of the close link between stemness, drug resistance and metastasis in various solid tumors [31–33] suggests that targeting CSCs or their stemness properties can complement cancer therapies. This study indicated that according to its suppressive function in NPC CSCs, *miR-183* might be a therapeutic target for the development of new treatment strategies.

In addition to that of *miR-183*, overexpression of *miR-96* transiently inhibited both anchorage-dependent and anchorage-independent tumor cell growth (Fig. 2). However, the growth inhibitory effect was lost in the C666-1 cells stably expressing *miR-96* (Fig. 3b). Despite its inhibitory effect on in vitro tumor sphere formation, the cells stably expressing *miR-96* showed no significant suppression of tumor formation in the in vivo nude mice model (Fig. 4b). The findings indicate that *miR-96* and *miR-183* exert differential inhibitory effects in C666-1 cells. It is likely that *miR-96* plays a less important role in regulating CSC properties in NPC. As in our previous study [7], the formation of tumor spheres (anchorage-independent growth) was used to assess CSC properties among NPC cells. The diminished capacity of tumor sphere formation in the NPC cells expressing *miR-96* or *miR-183* suggested that these miRNAs exert a suppressive role on the sphere-forming subpopulation of cells (Fig. 3c). The overexpression of these miRNAs in C666-1 cells, which resulted in the failure to form “large” tumor spheres, may be due to the heterogeneous structure of the tumor spheres [34].

The diminished properties of NPC CSCs in the stable *miR-96*- or *miR-183-*expressing NPC cells were also indicated by the suppression of pluripotent stem cell transcription factors SOX2 and OCT4 (Fig. 4a). Thus, *prima facie*, the overexpression of *miR-96* and *miR-183* would affect NPC tumorigenesis via suppression of the stemness properties of NPC cells. Contrary to SOX2 and OCT4, the expression of BMI1 was not regulated by *miR-96* or *miR-183* (Fig. 4a). BMI1 is a downstream target of *miR-203* [35], which was also significantly downregulated in NPC CSCs (Additional file 1: Figure S1B). It is likely that the downregulation of multiple stemness-inhibitory miRNA including *miR-96*, *miR-183*, and *miR-203* targets various pluripotent stem cell transcription factors in NPC CSCs. The role of *miR-96* and *miR-183* in the regulation of NPC CSC properties was also supported by the finding of reduced NICD3 and NICD4 expression in C666-1 cells stably expressing *miR-96* and *miR-183* (Fig. 4a). The NOTCH3 signaling pathway is constitutively activated and regulated by CSC properties in EBV-associated NPC [21]. The activated NOTCH3 signal was shown not only to confer oncogenic effects but also cisplatin resistance in NPC cells [21]. These findings suggest that *miR-96* or *miR-183* modulates NPC CSC properties by suppressing the expression of pluripotent stem cell transcription factor and NOTCH signal activity.

## Conclusions

This study provides evidences supporting the stemness-inhibitor miRNA *miR-183* to be tumor suppressor in EBV-associated NPC. Its abilities to suppress CSC properties in vitro and to effectively reduce tumor growth in vivo shed light on its role as a potential therapeutic target. Nevertheless, further study of the stability and mode of delivery of *miR-183* is required to develop an efficacious miRNA-based therapeutic approach for patients with NPC.

## Abbreviations

CSCs, cancer stem-like cells; EBV, Epstein-Barr virus; FACS, fluorescence-activated cell sorting; miRNA, microRNA; NPC, nasopharyngeal carcinoma; PBS, phosphate-buffered saline; qRT-PCR, quantitative reverse transcription and polymerase chain reaction.

## Additional files

Additional file 1: Figure S1. (A) List of miRNAs downregulated in C666-1 sphere-forming cells as detected by microarray analysis. (B) Selected miRNA expressions in sphere-forming and parental C666-1 cells were detected by qRT-PCR analysis. Significant downregulation of *miR-200a*, *miR-96*, *miR-183*, and *miR-203* expression was found in the nasopharyngeal carcinoma cancer stem-like cells. Student’s *t*-test was used to determine statistical significance between the two groups (*n* = 3, ***P* < 0.01, ****P* < 0.001). (ODP 150 kb)
