# Supplementary figures and images for: MicroRNA-183 suppresses cancer stem-like cell properties in EBV-associated nasopharyngeal carcinoma
Source: BMC Cancer. 2016 Jul 19;16:495. doi: 10.1186/s12885-016-2525-5 (PMC4950376; doi:10.1186/s12885-016-2525-5)

## Slide: page1
Supplementary figure 1

(A)
(B)

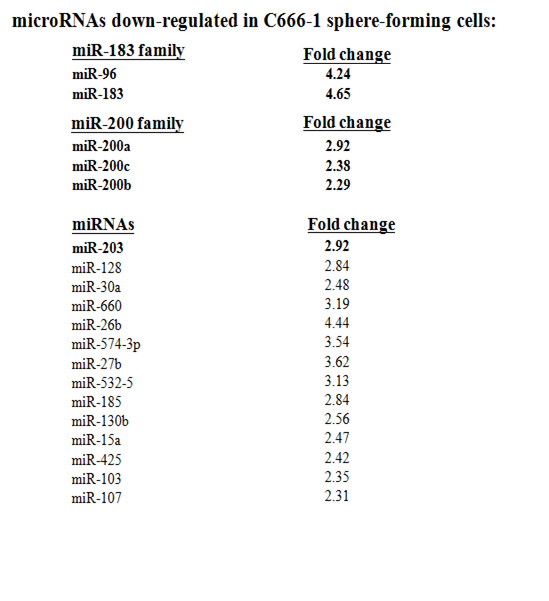

Supplement: Additional file 1: Figure S1. — (A) List of miRNAs downregulated in C666-1 sphere-forming cells as detected by microarray analysis. (B) Selected miRNA expressions in sphere-forming and parental C666-1 cells were detected by qRT-PCR analysis. Significant downregulation of miR-200a, miR-96, miR-183, and miR-203 expression was found in the nasopharyngeal carcinoma cancer stem-like cells. Student’s t-test was used to determine statistical significance between the two groups (n = 3, **P < 0.01, ***P < 0.001). (ODP 150 kb) [file 12885_2016_2525_MOESM1_ESM.odp]
